# Changes in lifestyle, adiposity, and cardiometabolic markers among young adults in Sweden during the COVID-19 pandemic

**DOI:** 10.1186/s12889-023-15998-w

**Published:** 2023-05-31

**Authors:** Sandra Ekström, Niklas Andersson, Inger Kull, Antonios Georgelis, Petter L. S. Ljungman, Erik Melén, Anna Bergström

**Affiliations:** 1 Center for Occupational and Environmental Medicine, Torsplan,Solnavägen 4, 113 65 Stockholm, Sweden; 2 Institute of Environmental Medicine, Karolinska Institutet, Box 210, 171 77 Stockholm, Sweden; 3 Department of Clinical Science and Education, Södersjukhuset, Karolinska Institutet, 118 83 Stockholm, Sweden; 4 Sachs’ Children and Youth Hospital, Södersjukhuset, 118 61 Stockholm, Sweden; 5 Department of Cardiology, Danderyd University Hospital, 182 57 Danderyd, Sweden

**Keywords:** Adiposity, Blood pressure, Lifestyle modifications, Adults

## Abstract

**Background:**

The COVID-19 pandemic has impacted on public health in several ways. The aim of the study was to investigate changes in lifestyle, adiposity, and cardiometabolic markers among young adults in Sweden during the COVID-19 pandemic and their determinants.

**Methods:**

The study included 1 004 participants from the population-based birth cohort BAMSE. Anthropometrics, body composition (bioelectric impedance analyses), pulse, and blood pressure were measured before (December 2016–May 2019; mean age 22.6 years) and during (October 2020–June 2021; mean age 25.7 years) the COVID-19 pandemic. Lifestyle changes during the pandemic were assessed through a questionnaire.

**Results:**

All measures of adiposity (weight, BMI, body fat percentage, trunk fat percentage) and cardiometabolic markers (blood pressure, pulse) increased during the study period (e.g., body fat percentage by a median of + 0.8% in females, *p* < 0.001, and + 1.5% in males, *p* < 0.001). Male sex, non-Scandinavian ethnicity, BMI status (underweight and obesity), and changes in lifestyle factors, e.g., decreased physical activity during the pandemic, were associated with higher increase in BMI and/or adiposity.

**Conclusion:**

Lifestyle factors, adiposity and cardiometabolic markers may have been adversely affected among young adults in Sweden during the COVID-19 pandemic compared with the preceding years. Targeted public health measures to reduce obesity and improve healthy lifestyle are important to prevent future non-communicable diseases.

**Supplementary Information:**

The online version contains supplementary material available at 10.1186/s12889-023-15998-w.

## Introduction

Obesity is one of the most pressing public health challenges globally and increases the risk of various non-communicable diseases including cardiovascular disease, type 2 diabetes, and several forms of cancer [1]. In Sweden, the prevalence of overweight and obesity has increased in recent decades, and it is estimated that more than half of the adult population and almost one in three young adults (16–29 years) suffers from overweight or obesity [2].

The COVID-19 pandemic has impacted on public health in several ways. The preventive measures taken to reduce the spread of COVID-19 affected daily life and activities during the pandemic. Studies have indicated that levels of physical activity decreased whereas sedentary time, weight status, blood pressure, and type 2 diabetes mellitus increased during this period [3–8]. The rate of increase in body mass index (BMI) was shown to be higher during the pandemic compared with the years before, among both US children [9, 10] and adults [11]. These trends seem to be more pronounced among individuals with established overweight and low education [4, 5]. A systematic review and meta-analysis on changes in bodyweight among adults during the first lockdown period found that body weight increased among 11.1% to 72% of the individuals in the included studies, with a weighted mean increase of 1.57 kg [12]. In children, weight gain has been reported to continue to increase after lockdown measures were eased, suggesting that the pandemic may have caused long-term adverse health consequences that are difficult to reverse [13].

Although BMI is a relatively valid measure of overweight or obesity at the group level, it cannot be used to differentiate between lean and fat mass. For some individuals, the pandemic may have led to increased time or opportunity for physical activity, which may result in an increased lean mass. For others, the pandemic may have resulted in less physical activity. Relatively few studies have investigated how the COVID-19 pandemic has influenced body composition [14–16] or cardiometabolic markers [17] and these have mostly been performed in small, selected populations [14–16]. One study on 1 669 boys in Czechia [18] observed that skeletal muscle mass decreased after physical activity restrictions, although no difference was found in body mass. Another study found no changes in body composition in healthy women before and after COVID-19 lockdown in Spain [16].

The potential impact of the COVID-19 pandemic is also likely to vary between countries due to different restrictions in different countries. In Sweden, society was relatively open during the pandemic, with no formal lockdown implemented. Certain restrictions, e.g., regarding maximum number of participants in public meetings and restaurants visits, were implemented. However, most measures, e.g., staying at home in case of symptoms of COVID-19 and social distancing, were based on voluntary participation. Still, a recent study showed that overweight and obesity increased among 3- and 4-year-old children in Sweden, especially in areas with lower socioeconomic status [19].

The aim of the present study was to investigate potential changes in lifestyle factors, BMI, overweight, body composition (% total body fat and trunk fat), blood pressure, and pulse among young adults in Sweden during the COVID-19 pandemic and to investigate determinants for these potential changes.

## Materials and methods

### Study population and study design

The study population includes participants from the population-based prospective birth cohort BAMSE, previously described in detail [20]. The BAMSE cohort originally included 4 089 newborns from Stockholm, Sweden, who have been followed up to young adulthood with repeated questionnaires and clinical examinations.

In November 2016–May 2019, a 24-year follow-up of the cohort was conducted with 75% of the participants answering a questionnaire (*n* = 3 064) and 56% participating in a clinical examination (December 2016–May 2019) including blood sampling and measurements of height, weight, body composition, pulse, and blood pressure [21].

In August 2020, a new follow-up of the BAMSE cohort was initiated, focusing on long-term effects of COVID-19 (referred to as the COVID-19 follow-up) [22, 23]. All 2 270 participants who completed the clinical examination at the 24-year follow-up were invited to the study, which consisted of three parts. In phase 1 (August–November 2020), participants answered a web-based questionnaire (questionnaire 1) covering self-reported symptoms of COVID-19, lifestyle, and sociodemographic factors (*n* = 1 644, 72% of invited). Phase 2 was conducted between October 2020 and June 2021 and included a clinical examination with blood sampling and measurements of height, weight, body composition, pulse, and blood pressure, as in the 24-year follow-up (*n* = 1 028). During the clinical visit, the participants answered a second questionnaire (questionnaire 2) including updated information on COVID-19 symptoms, lifestyle, and sociodemographic factors. The third part of the study (phase 3) took part between October 2021 and February 2022. All participants who completed the 24-year questionnaire and had provided their e-mail address (*n* = 2 981) were invited to answer a questionnaire (questionnaire 3).This included questions on COVID-19 symptoms and lifestyle changes during the pandemic.

**Fig. 1:**
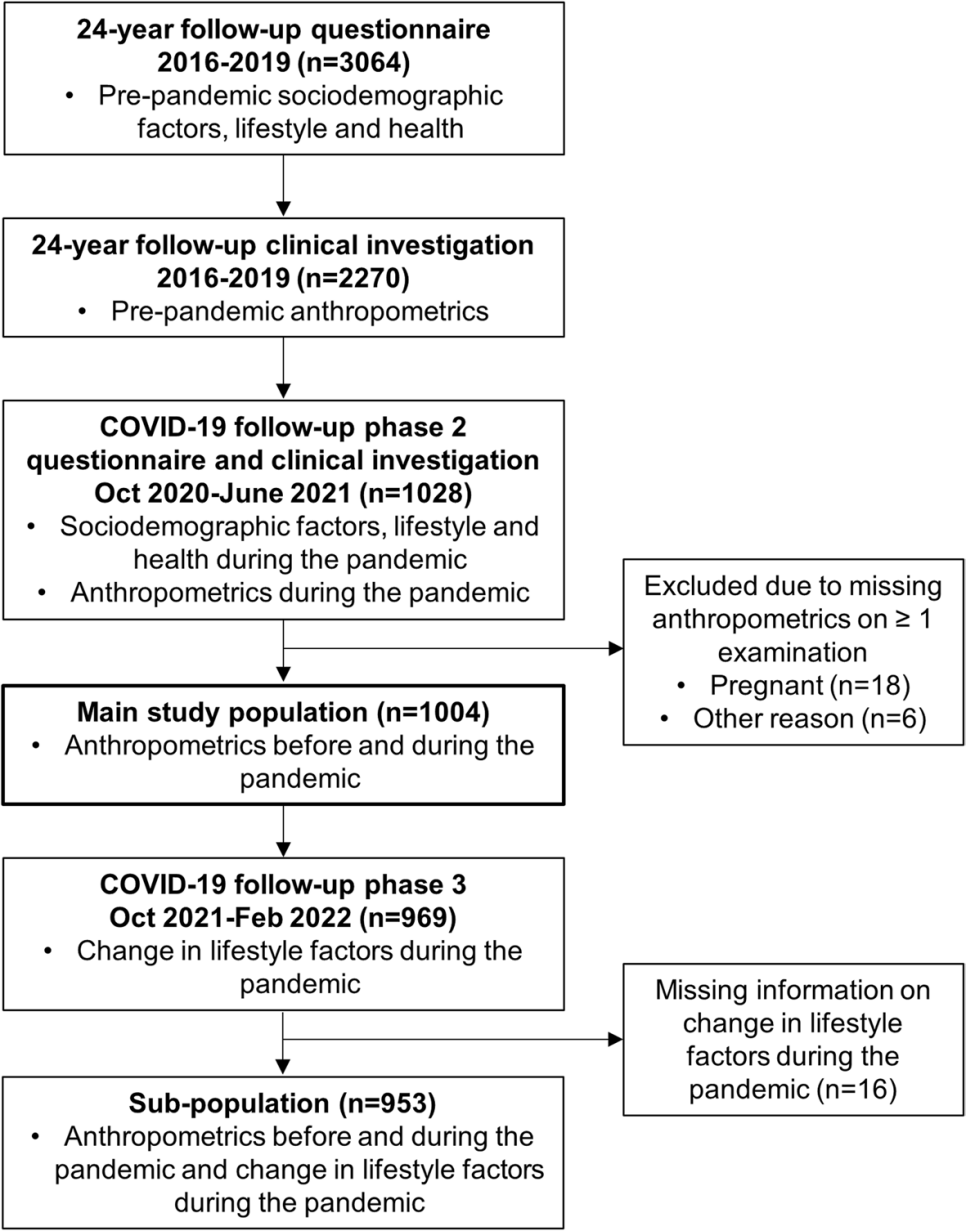
Overview of the data collection and inclusion of the participants in the study population

The study population of the present study include the BAMSE participants who took part in the clinical investigations at both the 24-year follow-up and the COVID-19 follow-up with information available on anthropometrics and body composition from both time points (*n* = 1 004). A sub-population included participants who also participated in the COVID-19 phase 3 follow-up and answered questions on lifestyle factors during the pandemic (*n* = 953). Pregnant women (*n* = 18) did not perform the body composition measurements and were not included in the study. An overview of data collection and inclusion of participants is shown in Fig. 1.

### Measurement of anthropometrics, body composition, and cardiometabolic markers

The examinations were performed by trained nurses with the same procedure used at the 24-year follow-up and the COVID-19 follow-up. Height was measured without shoes to the nearest 0.1 cm using a wall-mounted stadiometer. Height was measured twice at the 24-year examination and once at the COVID-19 examination. The first height measurement from the 24-year examination was used in the present analyses in order to make the two time points comparable. Weight and body composition (body fat percentage and trunk fat percentage) were assessed through bioelectric impedance analysis using a Tanita MC 780 body composition monitor in accordance with instructions from the manufacturer. BMI was calculated based on measured weight and height, analyzed as a continuous variable and categorized as underweight (< 18.5 kg/m^2^), normal weight (18.5–24.9 kg/m^2^), overweight (25–29.9 kg/m^2^), or obesity (≥ 30 kg/m^2^). Body fat percentage was used as a continuous variable and classified into high or normal using the pre-defined cut offs for “high”: ≥ 33% for females and ≥ 20% for males [24].

Resting blood pressure and pulse were measured using an automatic blood pressure meter (Omron HBP-1300). At each examination, three measurements were performed with the mean value used for analyses. Blood pressure was analyzed as a continuous variable and categorized as high if mean systolic blood pressure was ≥ 140 mmHg and/or mean diastolic blood pressure was ≥ 90 mmHg [25]. In the study population, 1 002 participants had blood pressure measured at both examination (all with three measurements from the COVID-19 examination; 996 had two measurements from the 24-year examination). Pulse data were available from 996 participants.

### Assessment of changes in lifestyle factors

Changes in lifestyle factors during the COVID-19 pandemic were assessed through the phase 3 questionnaire of the COVID-19 follow-up. Participants were asked about whether the following habits/health indicators had changed during the pandemic: physical activity, sedentary time, healthy dietary habits, alcohol intake, stress, sleep, and health. The response options were “decreased a lot,” “decreased,” “unchanged,” “increased,” and “increased a lot,”. In the analyses, the options “decreased a lot” and “decreased” were combined into “decreased” and the options “increased” and “increased a lot” into “increased.”

### Assessment of sociodemographic factors and other co-variables

Information on parental occupation was assessed in the baseline questionnaire when the participants were aged around 2–3 months and categorized as blue-collar or white-collar worker. Parental birth country was assessed in a parental questionnaire when the participants were around 8 years of age and categorized as outside of Scandinavia if any of the parents was born outside of Sweden, Norway, Denmark, or Finland. Education level, occupation status, smoking and snuff use were assessed in both the 24-year questionnaire and the COVID-19 follow-up phase 2 questionnaire (education level in phase 3). Living area was obtained from registered addresses at the time of the 24-year examination and the COVID-19 phase 3 follow-up and categorized as within or outside of Stockholm county. Education level was categorized as elementary school, high school, university/college < 3 years, or university/college ≥ 3 years. Occupation was categorized as studying, working or other (including unemployed, on parental leave, furloughed). Smoking and snuff use were categorized as yes (daily or occasional use) or no (ex-smoker/snuff user or never user).

Baseline physical activity and asthma were obtained through the 24-year questionnaire. Physical activity was categorized as high (≥ 7 h per week of moderate to vigorous activity or ≥ 3.5 h per week of vigorous activity) or low/moderate in accordance with International Physical activity Questionnaire guidelines [26]. Asthma was defined as doctor’s diagnosis of asthma (ever) together with symptoms of breathing difficulties or asthma medication occasionally or regularly in the preceding 12 months [27].

Previous COVID-19 disease was defined based on seropositivity against SARS-COV-2 immunoglobulin G (IgG), analyzed in blood samples from the COVID-19 examination in phase 2 (*n* = 32 individuals had been vaccinated against COVID-19 at this time point and were excluded).

### Statistical analyses

Normality of the continuous variables was tested using the Shapiro–Wilk test, stratified by sex. All variables except diastolic blood pressure in females departed significantly from the normal distribution. Continuous variables are therefore presented as median values with inter quartile ranges (IQRs). Non-parametric tests were used (the Wilcoxon matched-pairs signed-rank test for the paired data and the Wilcoxon rank-sum test or the Kruskal–Wallis test for unpaired data, as appropriate). For categorical variables, McNemar’s chi-squared test or the marginal homogeneity test were used for paired data and the chi-squared test for unpaired data). Associations between sociodemographic-/lifestyle factors and median change in BMI as well as body fat percentage before and during the COVID-19 pandemic were further analyzed in mutually adjusted quantile regression models [28].

All analyses were performed using the statistical software Stata version 16.1 (StataCorp LP, College Station. TX, USA). Missing data were handled with complete case analysis. A *p*-value < 0.05 was considered statistically significant.

## Results

### Description of the study population

The study population (*n* = 1 004) consisted of 624 (62.2%) females and 380 (37.8%) males. The median age was 22.5 years at the 24-year examination and 25.8 years at the COVID-19 examination (median time span 3.2 years). Education levels increased and the proportion who reported working as their main occupation increased during the study period, while the proportion of students decreased (Table 1).

**Table 1:** Sociodemographic and lifestyle characteristics before and during the COVID-19 pandemic (*n* = 1 004)

	**Before the pandemic** **(2016–2019)**^**1**^		**During the pandemic** **(2020–2021)**^**2**^		
**Continuous variables**	**Median**	**IQR**	**Median**	**IQR**	***p*-value**^**3**^
Age (years)	22.5	0.6	25.8	1.3	< 0.001
**Categorical variables**	**n**^**4**^	**%**	**n**^**4**^	**%**	***p*-value**^**5**^
**Living area**^**6**^					
Stockholm County	810	81.0	845	84.3	0.01
Outside of Stockholm County	190	19.0	157	15.7	
**Education**^**6**^					
Elementary school	26	2.6	14	1.5	< 0.001
High school	574	57.5	245	25.8	
University/college < 3 years	245	24.5	151	15.9	
University/college ≥ 3 years	154	15.4	541	56.9	
**Occupation**					
Studying	574	57.5	383	38.3	< 0.001
Working	361	36.0	557	55.6	
Other	65	6.5	61	6.1	
**Smoking**					
No	688	68.7	755	75.4	< 0.001
Ex-smoker	127	12.7	127	12.7	
Yes, sometimes	125	12.5	93	9.3	
Yes, every day	62	6.2	26	2.6	
**Snuff use**					
No	841	83.9	730	72.9	< 0.001
Ex-user	33	3.3	40	4.0	
Yes, sometimes	42	4.2	88	8.8	
Yes, every day	86	8.6	144	14.4	

*IQR* Inter quartile range

^1^Information obtained from the 24-year follow-up in 2016–2019

^2^Information obtained from the COVID-19 follow-up phase 2 iunless otherwise specified

^3^*p*-values obtained by using the Wilcoxon matched-pairs signed rank test

^4^Numbers may not add up to total due to missing data

^5^*p*-values obtained using the McNemar’s chi-squared test or the marginal homogeneity test

^6^During the pandemic, assessed in the COVID-19 follow-up phase 3, Oct 2021–Feb 2022

### Change in anthropometrics, adiposity and cardiometabolic markers during the pandemic

**Fig. 2:**
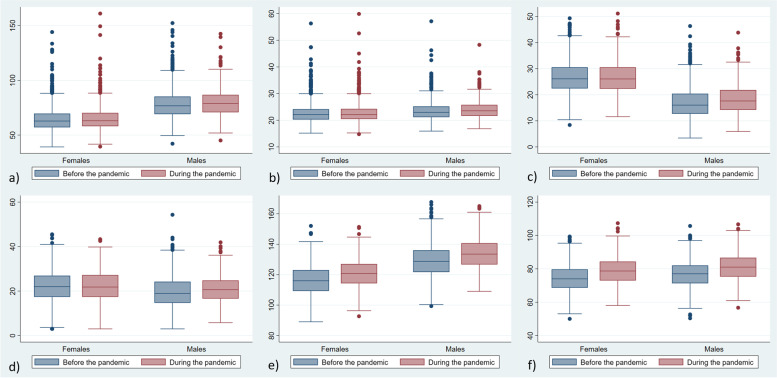
Distribution of anthropometrics and cardiometabolic markers before (2016–2019) and during (2020–2021) the COVID-19 pandemic (*n* = 1 004): a) weight (kg), b) BMI (kg/m^2^), c) total body fat (%), d) trunk fat (%), e) systolic blood pressure (mmHg), f) diastolic blood pressure (mmHg) in females and males. The boxes represent the 25^th^ – 75.^th^ percentile with the median value shown as horizontal lines, with outside values shown as dots. Differences in median values before vs. during the pandemic were tested with the Wilcoxon matched-pairs signed–rank test. All *p*-values < 0.001

**Fig. 3:**
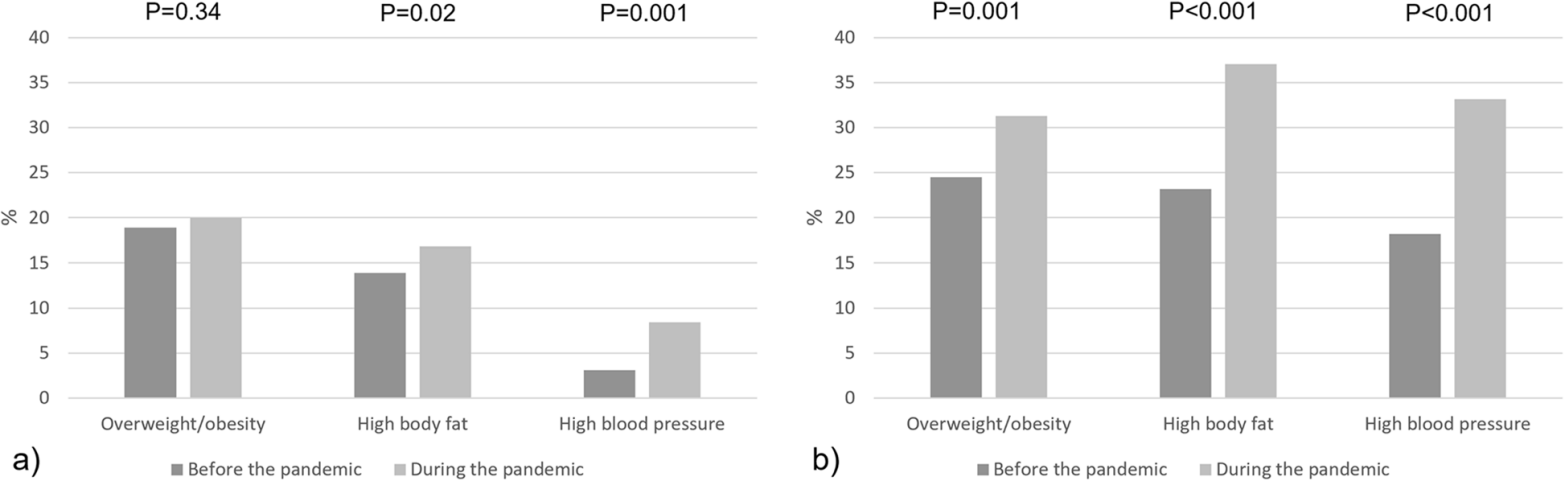
Change in BMI status, body fat percentage category and blood pressure category before and during the COVID-19 pandemic (*n* = 1 004) in **a**) females, **b**) males. Overweight: body mass index ≥ 25 kg/m^2^, high body fat percentage: ≥ 33% for females and ≥ 20% for males, high blood pressure: ≥ 140/90 mmHg. *P*-values were obtained using Mc Nemar’s chi-squared test

All measures of adiposity (weight, BMI, body fat percentage, trunk fat) increased during the study period in both sexes (Fig. 2 and Table S1). Weight and BMI increased by a median of 1.2 kg and 0.5 kg/m^2^, respectively, in females, and by 2.1 kg and 0.6 kg/m^2^, respectively, in males (all *p* < 0.001), whereas total body fat increased by a median of 0.8% in females (*p* < 0.001) and 1.5% in males (*p* < 0.001). In females, the prevalence of overweight/obesity did not change significantly (Fig. 2). However, analyses of individual categories showed that obesity increased from 3.2% to 7.1% (*p* < 0.001), whereas underweight, normal weight and overweight decreased (Table S2). In males, the prevalence of overweight/obesity increased from 23.2% to 37.1% (Fig. 3). The proportion of participants with high body fat percentage increased from 13.9% to 16.8% among females and 23.2% to 37.1% among males (Fig. 3 and Table S2).

Blood pressure (systolic and diastolic) as well as pulse increased during the study period in both sexes (Fig. 2 and Table S1). The proportion with high blood pressure increased from 3.1% to 8.4% in females (*p* < 0.001) and from 18.2% to 33.2% in males (*p* < 0.001), Fig. 3 and Table S2.

### Pre-pandemic factors associated with changes in BMI and body fat percentage

Table 2 shows changes in BMI and total body fat percentage in relation to pre-pandemic socioeconomic factors and other co-variables, stratified by sex. In females, median changes in BMI and body fat percentages were higher among participants with parents who were blue-collar workers at the time of inclusion at participant age 2–3 months, compared with those with white-collar worker parents (e.g., 0.95 kg/m^2^ vs. 0.43 kg/m^2^, *p* = 0.009). Median change in body fat percentage also differed in relation to pre-pandemic BMI status, where the highest increase was observed among females with underweight (2.85% compared with 0.70% in individuals with normal weight, 0.60% in females with overweight and 1.00% in females with obesity, *p* = 0.002). This pattern was not seen for median BMI change, which tended to be higher among females with obesity (1.36 kg/m^2^) than among normal weight females (0.36 kg/m^2^, *p* = 0.01). None of the other investigated factors (including asthma and previous COVID-19 disease, data not shown) were associated with changes in BMI or body fat percentage in females except snuff use which was associated with decreased BMI (*p* = 0.01) and body fat percentage (*p* = 0.02).

**Table 2:** Median change in body mass index (BMI) and total body fat percentage before and during the COVID-19 pandemic in relation to sociodemographic and lifestyle factors (*n* = 1 004)

	**Females (*n* = 624)**							**Males (*n* = 380)**						
		**BMI change (kg/m**^**2**^**)**			**Body fat change (%)**				**BMI change (kg/m**^**2**^**)**			**Body fat change (%)**		
	**n**^**1**^	**Median**	**IQR**	***P*-value**^**2**^	**Median**	**IQR**	***P*-value**	**n**^**1**^	**Median**	**IQR**	***P*-value**^**2**^	**Median**	**IQR**	***P*-value**^**2**^
**Parental occupation**^**3**^														
Blue-collar worker	86	0.95	2.19	0.009	1.70	5.00	0.01	51	0.63	2.81	0.89	0.70	5.90	0.25
White-collar worker	531	0.43	2.11		0.70	5.10		323	0.54	1.89		1.50	4.30	
**Parent born outside of Scandinavia**														
No	482	0.43	2.16	0.18	0.85	5.20	0.61	311	0.50	1.99	0.06	1.30	4.50	0.07
Yes	86	0.57	2.16		1.1	5.10		42	1.01	2.92		2.30	3.80	
**Education**^**4**^														
Elementary school/high school	357	0.56	2.30	0.10	1.10	5.30	0.07	243	0.67	2.04	0.31	1.60	3.80	0.47
University/college	265	0.40	1.98		0.60	5.00		134	0.38	2.02		1.25	4.00	
**Occupation**^**4**^														
Studying	365	0.40	2.07	0.46	0.60	5.00	0.08	211	0.56	2.45	0.73	1.50	5.00	0.73
Working	226	0.50	2.10		1.05	4.90		135	0.57	1.49		1.50	3.50	
Other	32	1.02	2.78		0.95	6.55		33	0.72	2.39		0.70	5.60	
**BMI status**^**4**^														
Underweight	38	0.78	1.57	0.01	2.85	4.00	0.002	16	1.14	1.29	0.10	3.00	5.25	0.046
Normal weight	469	0.36	1.98		0.70	5.30		271	0.57	2.05		1.50	4.50	
Overweight	97	0.84	3.10		0.60	5.30		79	0.33	2.37		0.80	4.70	
Obese	20	1.36	5.50		1.00	5.70		14	0.88	3.93		0.90	3.40	
**Smoking**^**4**^														
No	498	0.46	2.05	0.32	0.80	5.10	0.46	317	0.60	1.97	0.10	1.40	4.10	0.11
Yes	125	0.48	2.53		1.00	5.40		62	0.64	2.28		2.05	5.30	
**Snuff use**^**4**^														
No	583	0.48	2.09	0.01	0.90	5.00	0.02	291	0.54	2.20	0.24	1.40	4.70	0.20
Yes	40	-0.42	3.60		-1.30	8.30		88	0.75	1.67		2.10	3.85	
**Physical activity level**^**4**^														
Low/moderate	256	0.47	2.17	0.95	0.75	4.60	0.42	123	0.77	2.19	0.17	1.40	4.50	0.78
High	269	0.36	2.08		1.00	5.30		204	0.50	1.87		1.40	4.30	

*IQR* Inter quartile range

^1^Numbers may not add up to total due to missing data

^2^*P*-values obtained by using the Wilcoxon rank-sum test or the Kruskal–Wallis test

^3^Assessed at inclusion around 2–3 months of age

^4^Assessed in the 24-year follow-up in 2016–2019

Among males, pre-pandemic BMI status was associated with median changes in body fat percentage in a similar pattern as for females (*p* = 0.046), with the highest increase among the underweight. There was also a tendency for higher median BMI increase among males with a parent born outside of Scandinavia (1.01 kg/m^2^ compared with 0.50 kg/m^2^ among those with a parent born within Scandinavia, *p* = 0.06), whereas no difference was observed in relation to parental occupation.

The multivariable mutually adjusted quantile regression models (Table 3) showed that male sex was associated with 0.83% higher median increase in total body fat percentage compared with female sex (*p* = 0.02). Moreover, having a parent born outside of Scandinavia (compared with parents born in Scandinavia) and obesity (compared with normal weight) were associated with 0.76 kg/m^2^ (*p* = 0.001) and 1.45 kg/m^2^ (*p* = 0.002) higher median increase in BMI, respectively. Underweight was also associated with higher increase in body fat percentage, but this was not observed for obesity. None of the other variables were significantly associated with median change in BMI or body fat percentage.

**Table 3:** Mutually adjusted associations between sociodemographic-/lifestyle factors and median change in body mass index (BMI) and body fat percentage before and during the COVID-19 pandemic (*n* = 741)

	**Median change BMI (kg/m**^**2**^**)**			**Median change body fat (%)**		
	**Adj Beta-coeficient**^**1**^	**95% CI**	***P*-value**	**Adj Beta-coeficient**^**1**^	**95% CI**	***P*-value**
Male sex	0.19	-0.14, 0.52	0.25	0.83	0.16, 1.51	0.02
Parental white-collar worker	-0.16	-0.63, 0.32	0.52	-0.10	-1.08, 0.88	0.84
Parent born outside of Scandinavia	0.76	0.30, 1.21	0.001	0.93	-0.00, 1.87	0.05
Low education^2^	0.14	-0.19, 0.47	0.40	0.33	-0.34, 1.01	0.32
Worker^2^	-0.06	-0.41, 0.29	0.72	0.37	-0.36, 1.09	0.32
Underweight^2^	0.64	-0.03, 1.30	0.06	1.57	0.19, 2.94	0.03
Overweight^2^	0.14	-0.27, 0.57	0.49	-0.10	-0.97, 0.77	0.82
Obesity^2^	1.45	0.52, 2.39	0.002	0.37	-1.57, 2.30	0.71
Smoking^2^	0.28	-0.14, 0.70	0.20	0.80	-0.07, 1.67	0.07
Snuff use^2^	-0.07	-0.56, 0.42	0.77	-0.30	-1.31, 0.71	0.56
High physical activity^2^	-0.09	-0.41, 0.22	0.56	0.17	-0.48, 0.82	0.62

^1^Analyzed using multivariable mutually adjusted quantile regression models

^2^Assessed in the 24-year follow-up in 2016–2019

### Lifestyle changes during the COVID-19 pandemic

Self-reported lifestyle changes during the pandemic are presented in Table S3. For many of the lifestyle factors, a majority of participants reported that they had changed their habits in some direction. In total, 38.6% had decreased their physical activity levels during the pandemic (41.7% in females, 33.4% in males, *p* = 0.04), whereas 21.8% had increased their physical activity levels. A majority (57.8%) reported increased sedentary time during the pandemic, whereas dietary habits, sleep and overall health were unchanged for the majority. Reduced alcohol intake was reported by 44.1%, and 37.4% reported increased stress during the pandemic.

### Changes in BMI and body fat in relation to changes in lifestyle factors during the COVID-19 pandemic

In females, median change in BMI was related to changes in physical activity, dietary habits and health during the COVID-19 pandemic (Table 4). These factors, as well as sedentary time were also related to median change in body fat percentage. For example, females who increased their physical activity during the pandemic (*n* = 127) had reduced their body fat percentage by a median of 0.10%, whereas females who reduced their physical activity increased by a median of 1.10%. The same patterns were observed among males although only significant for changes in physical activity. Changes in alcohol intake, stress, or sleep were not related to changes in BMI or body fat percentage during the pandemic in either sex (Table 4).

**Table 4:** Median change in body mass index (BMI) and total body fat percentage before and during the COVID-19 pandemic in relation to self-reported change in lifestyle/health during the pandemic (*n* = 953)

	**Females**							**Males**						
		**BMI change (kg/m**^**2**^**)**			**Body fat change (%)**				**BMI change (kg/m**^**2**^**)**			**Body fat change (%)**		
	**n**^**1**^	**Median**	**IQR**	***P*-value**^**2**^	**Median**	**IQR**	***P*-value**^**2**^	**n**^**1**^	**Median**	**IQR**	***P*-value**^**2**^	**Median**	**IQR**	***P*-value**^**2**^
**Physical activity**														
Reduced	250	0.64	2.16	0.02	1.10	4.90	0.003	118	0.94	2.19	0.04	2.05	4.40	0.03
Unchanged	223	0.48	2.07		1.00	5.40		154	0.44	1.81		1.40	4.60	
Increased	127	0.17	2.53		-0.10	5.70		81	0.22	1.61		1.00	3.80	
**Sedentary time**														
Reduced	52	0.06	2.18	0.11	-0.15	4.85	0.048	20	0.89	1.74	0.75	1.95	4.05	0.42
Unchanged	212	0.21	2.08		0.75	5.45		129	0.47	1.70		1.10	4.30	
Increased	332	0.63	2.17		1.10	4.90		204	0.58	2.17		1.60	4.40	
**Healthy dietary habits**														
Reduced	135	0.99	2.03	0.001	1.60	4.10	0.001	72	0.99	2.71	0.047	1.55	5.20	0.74
Unchanged	359	0.29	2.10		0.60	6.00		215	0.42	1.89		1.40	4.10	
Increased	105	0.21	2.25		0.30	5.60		66	0.55	1.75		1.40	3.90	
**Alcohol intake**														
Reduced	261	0.57	2.21	0.33	0.80	5.20	0.50	158	0.41	2.13	0.27	1.35	4.50	0.71
Unchanged	263	0.38	2.13		0.90	5.40		148	0.70	1.99		1.45	4.40	
Increased	76	0.27	2.05		0.95	5.00		45	0.37	1.57		1.30	5.00	
**Stress**														
Reduced	80	0.32	2.13	0.75	0.70	5.05	0.99	41	0.50	2.13	0.30	1.60	4.80	0.40
Unchanged	272	0.47	1.97		0.90	4.95		204	0.47	1.87		1.40	4.05	
Increased	248	0.48	2.20		0.85	5.60		108	0.70	2.30		1.75	4.55	
**Sleep**														
Reduced	84	0.72	1.89	0.71	1.15	5.70	0.50	59	0.70	2.43	0.54	1.30	5.40	0.90
Unchanged	360	0.47	2.13		0.75	5.20		227	0.50	1.87		1.40	4.40	
Increased	156	0.34	2.16		0.90	5.20		67	0.55	1.98		1.50	3.80	
**Health**														
Reduced	177	0.77	2.03	0.04	1.50	4.40	0.002	80	0.76	2.38	0.15	1.75	5.45	0.12
Unchanged	320	0.42	2.08		0.85	4.80		201	0.56	1.74		1.50	3.90	
Increased	102	0.04	2.61		-0.90	6.60		72	0.15	2.12		1.10	3.90	

*IQR* inter quartile range

^1^Numbers may not add up to total due to missing data

^2^*P*-values obtained using the Kruskal Wallis test

## Discussion

This study, based on data from 1 004 Swedish young adults from a population-based cohort, showed that BMI, body fat percentage, blood pressure, and pulse increased from the pre-pandemic period in 2016–2019 until the pandemic period in 2020–2021. Male sex, having a parent born outside of Scandinavia, and BMI status (underweight and obesity), were associated with higher increase in BMI and/or adiposity. A large proportion of the participants reported changes in lifestyle factors during the COVID-19 pandemic, including reduced physical activity and increased sedentary time. Self-reported lifestyle changes during the pandemic were associated with changes in adiposity, with physical activity, sedentary time and dietary habits identified as the most important factors.

Our results are in line with previous studies which have observed increases in BMI and obesity prevalence during the COVID-19 pandemic [8, 10, 28–31]. The results are also in line with one large retrospective study in China observing that cardiometabolic profiles were negatively affected by the pandemic [17]. For blood pressure, a review in children and adolescents found mixed results, with two studies showing increases in blood pressure levels, whereas one showed a relevant reduction [8].

The impact of the COVID-19 pandemic on public health is likely to vary between countries due to differing restrictions. In the present study, we observed significant increases in all measures of adiposity and blood pressure, despite the fact that Swedish society remained relatively open during the pandemic. Large increases in blood pressure were observed, with a particularly high prevalence of high blood pressure among males (from 18 to 33% during the study period). These numbers are higher than those of other studies, e.g., the US National Health and Nutrition Examination Survey 2015–2016, which observed that 9.2% of men and 5.6% of women aged 18–39 years were classified as having high blood pressure when the same methods and definition as in our study were used [32]. The prevalence of hypertension increases with age, and in Sweden, 25% of females and 36% of males aged 30–79 years were estimated to have high blood pressure in 2019 according to a large, pooled analysis of studies representative of the population [33]. However, no published representative data on young adults in Sweden were found to compare our results with.

We observed that male sex, parental origin outside of Scandinavia, underweight, and obesity were associated with higher increase in BMI and/or body fat. Previous studies have shown that lower education [4, 5], higher BMI [5] and ethnicity were associated with larger weight gain or decreased healthy habits during the pandemic, with greater increases in Black and Hispanic US children [28, 31]. Although this study did not reveal any significant differences in BMI change related to education, the overall evidence suggests that the pandemic has disproportionally affected people with lower socioeconomic status, not only in terms of disease severity, but also in regard to indirect health consequences.

The results regarding self-reported lifestyle changes are also in line with most previous studies suggesting a negative impact of the COVID-19 pandemic on lifestyle behaviours such as lower levels of physical activity and increased sedentary time [5, 29, 30, 34]. This is also supported by objective data from Fitbit Inc©, showing a reduction in step counts of 9% in Sweden and 7–38% in European countries during the week ending March 22, 2020 compared with during the same week in the year before [35]. Several studies have shown decreases in healthy dietary habits during the pandemic [5, 34, 36], although more home-cooked meals and lower consumption of fried foods have also been reported [3]. However, the direction of lifestyle changes may vary between groups and countries [37–39], which was also seen in this study (e.g., 22% reported having increased their physical activity).

The strengths of the present study include the large sample size, the population-based design, and the repeated measurements of BMI, body composition, and blood pressure. Limitations include the relatively wide time frame of the pre-pandemic measurements from December 2016–May 2019. Therefore, it is not possible to determine if the increases in adiposity occurred during the pandemic or before. The different phases of the pandemic with various restrictions and recommendations may also have affected people’s lifestyle in different ways. As information on lifestyle changes was collected relatively late in the pandemic (October 2021–February 2022), this study may have underestimated changes during the early phase of the pandemic when stricter restrictions were generally imposed, and no SARS-CoV-2 vaccination was yet available. Further, it is not possible to know whether the changes seen are attributable to the pandemic or other factors, although we observed that reported lifestyle changes during the pandemic were related to changes in adiposity. It is known that BMI increases with age, also after childhood, and BMI seems to continue to increase over time [2, 40]. A longitudinal study investigating post-pubertal development of BMI and body fat percentage in 107 Swedish men followed from 1994 (at a mean age of 17.1 years) observed a weight gain from 80.9 kg to 82.3 kg between follow-ups conducted at on average 22.8 years and 24.8 years, similar to the ages of our examinations [41]. This corresponded to an increase in BMI of 1.4 kg/m^2^, which is slightly higher than seen in our study. However, that study included active and former athletes, with the latter having a greater BMI increase than the non-athletes [41]. Other limitations include the potential for selection bias, as the present study included only around one fourth of the original cohort. This may have underestimated the adverse changes in lifestyle factors, adiposity and cardiometabolic markers as the drop-out rate may be higher in less health-conscious individuals. Another weakness in the present study is that high blood pressure was defined based on a single examination visit, although three measurements with similar values were taken for the majority of participants. In addition, there was no information on change in physical activity intensity or duration and no detailed information on type of dietary changes.

## Conclusions

In conclusion, lifestyle factors, adiposity and cardiometabolic markers may have been adversely affected among young adults in Sweden during the COVID-19 pandemic, compared with the preceding years. Although it is unknown whether these changes were due to the pandemic, we believe that they deserve attention and appropriate actions. Public health measures to increase the prevalence of a healthy lifestyle, preferably targeted at vulnerable groups, are important in order to reduce obesity among young adults and prevent future non-communicable diseases.

## Supplementary Information

**Additional file 1.** 

## Data Availability

The datasets generated and/or analyzed during the current study are not publicly available due to the dataset containing sensitive personal data but are available from the corresponding author on reasonable request and with permission from Karolinska Institutet.
