# Supplementary material for: Changes in lifestyle, adiposity, and cardiometabolic markers among young adults in Sweden during the COVID-19 pandemic
Source: BMC Public Health. 2023 May 31;23:1026. doi: 10.1186/s12889-023-15998-w (PMC10230124; doi:10.1186/s12889-023-15998-w)
Supplement: Supplementary file 1 — Additional file 1. [file 12889_2023_15998_MOESM1_ESM.docx]

# **Changes in lifestyle, adiposity, and cardiometabolic markers among young adults in Sweden during the COVID-19 pandemic**

# **Supplementary information**

Sandra Ekström^1,2,3^, Niklas Andersson^2^, Inger Kull^3,4^, Antonios Georgelis^1^, Petter LS Ljungman^2,5^, Erik Melén^2,3,4^, Anna Bergström^1,2^

1. Center for Occupational and Environmental Medicine, Region Stockholm, Stockholm, Sweden

2. Institute of Environmental Medicine, Karolinska Institutet, Stockholm, Sweden

3. Department of Clinical Science and Education Södersjukhuset, Karolinska Institutet, Stockholm, Sweden

4. Sachs' Children and Youth Hospital, Södersjukhuset, Stockholm, Sweden

5. Department of Cardiology, Danderyd University Hospital, Danderyd, Sweden.

# Additional file 1: **Table S1.** Anthropometrics and cardiometabolic markers before and during the COVID-19 pandemic (n=1 004). **Table S2.** Change in BMI status, body fat percentage category and blood pressure category before and during the COVID-19 pandemic (n=1 004). **Table S3.** Self-reported changes in lifestyle factors during the COVID-19 pandemic among participants in the sub-population

# file format: .pdf

# title of data: Changes in lifestyle, adiposity, and cardiometabolic markers among young adults in Sweden during the COVID-19 pandemic - Supplementary information.

# Description of data: supplementary information for the manuscript entitled “Changes in lifestyle, adiposity, and cardiometabolic markers among young adults in Sweden during the COVID-19 pandemic”.

**Table S1.** Anthropometrics and cardiometabolic markers before and during the COVID-19 pandemic (n=1 004)

|  | **Females (n=624)** | | | | | | | **Males (n=380)** | | | | | | |
| --- | --- | --- | --- | --- | --- | --- | --- | --- | --- | --- | --- | --- | --- | --- |
|  | **Before the pandemic (2016-2019)** | | **During the pandemic**  **(2020-2021)** | |  | **Individual change during vs before the pandemic** | | **Before the pandemic (2016-2019)** | | **During the pandemic**  **(2020-2021)** | |  | **Individual change during vs before the pandemic** | |
| **Continuous variables** | **Median** | **IQR** | **Median** | **IQR** | **P-value^1^** | **Median** | **IQR** | **Median** | **IQR** | **Median** | **IQR** | **P-value^1^** | **Median** | **IQR** |
| Height (cm) | 168.9 | 8.0 | 169.0 | 8.5 | <0.001 | -0.1 | 0.9 | 182.1 | 10.7 | 182.0 | 11 | 0.19 | 0.0 | 0.9 |
| Weight (kg) | 62.4 | 12.6 | 63.0 | 12.3 | <0.001 | 1.2 | 5.9 | 76.1 | 15.9 | 78.7 | 16.1 | <0.001 | 2.1 | 6.8 |
| Body mass index, BMI (kg/m^2^) | 21.9 | 3.8 | 22.2 | 3.8 | <0.001 | 0.5 | 2.1 | 22.8 | 3.48 | 23.5 | 4.1 | <0.001 | 0.6 | 2.0 |
| Total body fat (%) | 25.6 | 7.5 | 26.0 | 8.3 | <0.001 | 0.8 | 5.2 | 15.5 | 7.0 | 17.6 | 7.6 | <0.001 | 1.5 | 4.5 |
| Trunk fat (%) | 21.6 | 9.4 | 21.7 | 9.8 | <0.001 | 0.8 | 5.7 | 18.5 | 9 | 20.6 | 8.2 | <0.001 | 1.5 | 5.2 |
| Systolic blood pressure (mmHg)^3^ | 116.2 | 13.3 | 120.7 | 13.0 | <0.001 | 3.7 | 11.3 | 129.0 | 14.3 | 133.3 | 13.8 | <0.001 | 4.0 | 11.8 |
| Diastolic blood pressure (mmHg)^3^ | 74.0 | 11.2 | 78.7 | 11.3 | <0.001 | 4.2 | 11 | 77.0 | 10.2 | 81.0 | 11.3 | <0.001 | 3.7 | 10.8 |
| Pulse (bpm)^4^ | 73.3 | 15.7 | 74.7 | 16.0 | 0.024 | 1.3 | 14 | 73.3 | 15.7 | 75.3 | 17.0 | 0.072 | 2.0 | 18 |

IQR: Inter quartile range.

^1^ p-values obtained using the Wilcoxon matched-pairs signed-rank test.

^3^ n = 1002, 622 females 380 males.

^4^ n = 996, 619 females, 377 males.

|  | **Females (n=624)** | | | | | **Males (n=380)** | | | | |
| --- | --- | --- | --- | --- | --- | --- | --- | --- | --- | --- |
|  | **Before the pandemic (2016-2019)** | | **During the pandemic**  **(2020-2021)** | |  | **Before the pandemic (2016-2019)** | | **During the pandemic**  **(2020-2021)** | |  |
|  | **n** | **%** | **n** | **%** | **P-value** | **n** | **%** | **n** | **%** | **P-value** |
| Underweight (<18.5 kg/m^2^) | 38 | 6.1 | 36 | 5.8 | <0.001**^1^** | 16 | 4.2 | 8 | 2.1 | <0.001**^1^** |
| Normal weight (18.5–24.9 kg/m^2^) | 468 | 75.0 | 463 | 74.2 |  | 271 | 71.3 | 253 | 66.2 |  |
| Overweight (≥25 kg/m^2^) | 98 | 15.7 | 81 | 13.0 |  | 79 | 20.8 | 97 | 25.5 |  |
| Obesity (≥30 kg/m^2^) | 20 | 3.2 | 44 | 7.1 |  | 14 | 3.7 | 22 | 5.8 |  |
| High body fat (≥33% for females and ≥20% for males) | 87 | 13.9 | 105 | 16.8 | 0.02**^2^** | 88 | 23.2 | 141 | 37.1 | <0.001**^2^** |
| High blood pressure (≥140/90 mmHg) | 19 | 3.1 | 52 | 8.4 | <0.001**^2^** | 69 | 18.2 | 126 | 33.2 | <0.001**^2^** |

**Table S2.** Change in BMI status, body fat percentage category and blood pressure category before and during the COVID-19 pandemic (n=1 004)

^1^ p-values obtained using the marginal homogeneity test.

^2^ p-values obtained using the McNemar’s chi-squared test.

^3^ n = 1002, 622 females 380 males.

^4^ n = 996, 619 females, 377 males.

| **Table S3.** Self-reported changes in lifestyle factors during the COVID-19 pandemic^1^ among participants in the sub-population | | | | |
| --- | --- | --- | --- | --- |
|  | **Females** | **Males** | **Total** |  |
|  | **n (%)** | **n (%)** | **n (%)** | **P-value^2^** |
| **Physical activity (n=953)**   - Reduced - Unchanged - Increased | 250 (41.7)  223 (37.2)  127 (21.2) | 118 (33.4)  154 (43.6)  81 (23.0) | 368 (38.6)  377 (39.6)  208 (21.8) | 0.04 |
| **Sedentary time (n=949)**   - Reduced - Unchanged - Increased | 52 (8.7)  212 (35.6)  332 (55.7) | 20 (5.7)  129 (36.5)  204 (57.8) | 72 (5.6)  341 (35.9)  204 (57.8) | 0.23 |
| **Healthy dietary habits (n=952**)   - Reduced - Unchanged - Increased | 135 (22.5)  359 (59.9)  105 (17.5) | 72 (20.4)  215 (60.9)  66 (18.7) | 207 (21.7)  574 (60.3)  171 (18.0) | 0.72 |
| **Alcohol intake (n=951)**   - Reduced - Unchanged - Increased | 261 (43.5)  263 (43.8)  76 (12.7) | 158 (45.0)  148 (42.2)  45 (12.8) | 419 (44.1)  411 (43.2)  121 (12.7) | 0.88 |
| **Stress (n=953)**   - Reduced - Unchanged - Increased | 80 (13.3)  272 (45.3)  248 (41.3) | 41 (11.6)  204 (57.8)  108 (30.6) | 121 (12.7)  476 (50.0)  356 (37.4) | 0.001 |
| **Sleep (n=953)**   - Reduced - Unchanged - Increased | 84 (14.0)  360 (60.0)  156 (26.0) | 59 (16.7)  227 (64.3)  67 (19.0) | 143 (15.0)  587 (61.6)  223 (23.4) | 0.04 |
| **Health (n=952)**   - Reduced - Unchanged - Increased | 177 (29.6)  320 (53.4)  102 (17.0) | 80 (22.7)  201 (56.9)  72 (20.4) | 257 (27.0)  521 (54.7)  174 (18.3) | 0.06 |

^1^ assessed in the COVID-19 follow-up phase 3 questionnaire in October 2021 – February 2022.

^2^ P-value obtained using the chi-squared test.
